# Brief version of Zarit Burden Interview (ZBI) for burden assessment in older caregivers

**DOI:** 10.1590/1980-57642018dn13-010015

**Published:** 2019

**Authors:** Aline Cristina Martins Gratão, Allan Gustavo Brigola, Ana Carolina Ottaviani, Bruna Moretti Luchesi, Érica Nestor Souza, Estefani Serafim Rossetti, Nathalia Alves de Oliveira, Marielli Terassi, Sofia Cristina Iost Pavarini

**Affiliations:** 1 PhD. Professors, Undergraduate Program in Gerontology, Universidade Federal de São Carlos, São Carlos, SP, Brazil.; 2 MSc. Candidate, Graduate Nursing Program, Universidade Federal de São Carlos, São Carlos, SP, Brazil.; 3 PhD. Professors, Universidade Federal do Mato Grosso do Sul - Três Lagoas, MS, Brazil.

**Keywords:** caregivers, older adult, psychological stress, validation studies, geriatric nursing, cuidadores, idoso, estresse psicológico, estudos de validação, enfermagem geriátrica

## Abstract

The degree of burden related to the demands of daily care provided to a dependent older adult is important to evaluate due to the negative influence exerted on caregivers’ physical and psychological health.

**Objective:**

To analyze the validity of the 12-item version of the Zarit Burden Interview administered to older caregivers of community-dwelling older dependent individuals and suggest a cut-off score based on quartiles.

**Methods:**

Three hundred and forty-one older caregivers (mean age: 69.6±7.1 years; 76.8% women) registered with primary healthcare centers were evaluated using the ZBI-12. Additional evaluations addressed stress (Perceived Stress Scale [PSS]), depressive symptoms (Geriatric Depression Scale [GDS]) in the older caregivers and the degree of dependence of the older care recipients (Lawton and Brody [L&B]).

**Results:**

Cronbach’s alpha demonstrated very good internal consistency (α=0.81). Correlations were found between all ZBI-12 items and overall score on the PSS (r=0.53; p<0.01). GDS (r=0.43; p<0.01) and L&B (r= -0.23; p<0.01) scale scores. The PSS demonstrated the strongest correlation with ZBI-12 score and proved to be the standard reference. Based on caregivers with a higher degree of stress considering the PSS score quartiles, a cut-off score of 13 points on the ZBI-12 is suggested for screening burden in community-dwelling older caregivers, but should not be assumed as normative data.

**Conclusion:**

The ZBI-12 can be considered valid for evaluation of burden in clinical practice and research as a fast, efficient option for screening burden among older caregivers of community-dwelling older adults.

## RESUMO.

O grau de sobrecarga relacionado às demandas do cuidado diário ao idoso dependente é uma importante medida a ser avaliada por influenciar de modo negativo na saúde física e psicológica do cuidador.

**Objetivo:**

Analisar a validade da Zarit Burden Interview versão 12 itens (ZBI-12) em idosos cuidadores de idosos da comunidade e sugerir nota de corte de acordo com quartis.

**Métodos:**

Foram avaliados 341 idosos cuidadores (M=69,6±7,1 anos; 76,8% feminino) cadastrados na Estratégia Saúde da Família utilizando a ZBI-12. Adicionalmente, foram realizadas avaliações de estresse (Escala de Estresse Percebido [PSS]) e de sintomas depressivos (Escala de Depressão Geriátrica [GDS]) no idoso cuidador, e avaliada a dependência dos idosos receptores de cuidado (Escala de Atividades da Vida Diária de Lawton e Brody [L&B]).

**Resultados:**

As análises por alfa de Cronbach mostraram consistência interna de α=0.81. Foi encontrada correlação entre todos os itens da ZBI-12 e do escore geral com os escores de PSS (r=0,53; p<0,01), GDS (r=0,43; p<0,01) e L&B (r= -0,23; p<0,01). Especificamente, a PSS obteve associação mais estreita com o escore da ZBI-12, se mostrando o padrão-referência. Considerando a divisão por quartil da PSS, para os cuidadores mais estressados, sugere-se nota de corte de 13 pontos na ZBI-12 para rastreio da sobrecarga em idosos cuidadores da comunidade em geral.

**Conclusão:**

A ZBI-12 apresentou boa consistência interna e pode ser considerada válida nessa população. Espera-se que o instrumento auxilie na avaliação da sobrecarga na prática clínica e na pesquisa e se torne uma alternativa de aplicação rápida e eficiente no rastreio de sobrecarga entre cuidadores mais velhos na comunidade.

Burden has been defined as the financial, physical and psychological consequence related to the responses and attitudes of a caregiver to the demands of providing care.^1^^-^ 3 It is considered a multidimensional concept that involves negative personal evaluations with regard to context, providing care and changes in one’s wellbeing.^1^ Considering the combined physical and emotional strain, burden can be expressed as constant fatigue, sleep disorders, muscle pain, loss of attention and concentration, impatience, anxiety, depression, irritability and constant tension.^2^^,^ 3 In contrast, caregivers with a lower degree of burden are more likely to report positive results related to wellbeing, better perceived health, fewer depressive symptoms and lower levels of stress.^4^^,^ 5

The research literature and meta-analysis results on studies conducted with informal caregivers of older adults, physical burden, financial burden, relationship tension and depressive symptoms were all greater in this group compared to non-caregivers.^6^ The high degrees of caregiver burden may be related to the increase in the severity of behavioral symptoms and dependence of the care recipient as well as insufficient social support and increased feelings of guilt and stress in the caregiver.^7^^,^ 8

The Zarit Caregiver Burden Interview (ZBI) is an assessment tool for evaluating caregiver burden that is widely used around the world. The ZBI was developed in 1980 to evaluate the perceived impact of providing care on aspects such as the caregiver’s health, personal and social life, financial situation, emotional wellbeing and interpersonal relationships.^9^ The original version in English had 29 items and was followed by a 22-item version (ZBI-22) in 1985.^10^ Although the ZBI-22 demonstrates excellent internal consistency, its application time and difficulties in understanding the items can affect the reliability of results. Thus, a study involving 413 caregivers of older adults with cognitive decline proposed the 12-item version (ZBI-12) for the evaluation of caregiver burden^11^ and has been translated into different languages, including Spanish, Japanese and Chinese.^12^^-^ 14 The 12-item version has been used in several studies involving caregivers of individuals with cognitive decline^15^^-^ 17 and its use in different contexts has also been proposed.^18^ In the international literature, a large number of studies have used the ZBI-12 to evaluate burden of caregivers in different care contexts and this measure has proved to be sensitive and effective for evaluating overall burden in caregivers of older adults. A study conducted with 270 caregivers of older adults with dementia in Taiwan to evaluate the psychometric properties of the ZBI-12 found that the measure assists in the evaluation of burden, the identification of associated factors and the establishment of interventions in a timely manner in both a clinical context and the community.^19^

Assessment tools are important for the evaluation of burden among caregivers of community-dwelling older adults with diverse comorbidities, as physiological ageing can contribute to an increase in the number of chronic diseases.^20^ Short versions facilitate the application of these assessment tools at healthcare services and in studies,^11^ especially in populations from middle and low-income countries. These versions also help support interventions and actions proposed by health professionals aimed at reducing burden, thereby contributing to the physical and psychological health of caregivers.

The aim of the present study was to analyze the internal consistency and validity of the ZBI-12 in caregivers of community-dwelling older adults and suggest a cut-off score based on standard reference assessment tools. The hypothesis is that the ZBI-12 has good internal consistency and is correlated with other assessment tools that address the psychological wellbeing of caregivers.

## METHODS

The present cross-sectional study was conducted in accordance with the Revised Standards for Quality Improvement Reporting Excellence (SQUIRE 2.0 - checklist) for the description of the results. The sample consisted of 341 persons who met the following inclusion criteria, aged 60 years or older; performed the role of caregiver of their elderly dependent family member living in the same household, and enrolled in one of the primary healthcare centers in the city of São Carlos, state of São Paulo, Brazil, located 235 km from the state capital. The exclusion criterion of the research was refusal by the participant to sign the Free and Informed Consent Form.

The degree of dependence of the elderly dependent on care was verified using the evaluation of the level of dependence for basic and instrumental activities of daily living analyzed by the Katz Index and Lawton and Brody’s Scale.

The data were collected by an oral interview, where carers with cognitive deficits were excluded with self-report. Therefore, interviewees who could understand and complete the data collection instruments were selected for the study.

The interview was conducted at the homes of the participants between April and November 2014 by students and health professionals who had undergone training for the administration of the following data collection instruments:

- Zarit Burden Interview: the ZBI-22 was developed by Zarit, Reever and Bach-Peterson in 1980 and was translated and validated for use in Brazil by Scazufca in 2002.^21^ The 22 items address the perceived impact of the act of providing care on the physical health, emotional health, social activities and financial situation of the caregiver. Each item has five response options ranging from “never” to “nearly always”. Twelve of these items were extracted for use in the present study, proposed by Bedard et al.^11^ and are listed in Chart 1.

**Chart 1:** Zarit Burden Interview Scale - 12-item version^11^ administered to older caregivers of older adults

ZBI-22 items	Instructions: Recommendations for the administration and scoring of each statement follow the original publication of the scale for the Brazilian context.^20^
2.	Do you feel that because of the time you spend with (care recipient) that you don't have enough time for yourself?
3.	Do you feel stressed between caring for (care recipient) and trying to meet other responsibilities for your family or work?
5.	Do you feel angry when you are around (care recipient)?
6.	Do you feel that (care recipient) currently affects your relationships with other family members or friends in a negative way?
9.	Do you feel strained when you are around (care recipient)?
10.	Do you feel your health has suffered because of your involvement with (care recipient)?
11.	Do you feel that you don't have as much privacy as you would like because of (care recipient)?
12.	Do you feel that your social life has suffered because you are caring for (care recipient)?
17.	Do you feel you have lost control of your life since (care recipient)'s illness?
19.	Do you feel uncertain about what to do about (care recipient)?
20.	Do you feel you should be doing more for (care recipient)?
22.	Overall, how burdened do you feel in caring for (care recipient)?

For the identification of an assessment tool that could serve as the standard reference, the following were also administered to the participants:

- Perceived Stress Scale (PSS): the PSS was translated and validated for use in older adults in Brazil.^22^ It contains 14 items that indicate the level of perceived stress in older adults. The total ranges from 0 to 57, with higher scores denoting a higher level of perceived stress.^22^
- Geriatric Depression Scale (GDS): the GDS was developed to screen mood disorders in older adults and has been translated and validated for use in Brazil.^23^ The 15-item scale was employed (GDS-15)^23^ where a score of 6 points or more indicates the presence of depressive symptoms and a score of 11 points or more characterizes severe depressive symptoms.
- Lawton and Brody (L&B) Instrumental Activities of Daily Living Scale:^24^ This was used to evaluate the degree of dependence of the care recipient. The final score ranges from 7 to 21 points, with a lower score denoting a greater degree of dependence. Satisfactory indices and good reliability were found during the adaptation of the scale to the Brazilian context and a score of 7 points indicates complete dependence, 8 to 20 points indicates partial dependence and 21 points, complete independence.^25^

This study was conducted in accordance with all ethical precepts that govern research involving human subjects and received approval from the Human Research Ethics Committee of the *Universidade Federal de São Carlos* (certificate numbers: 416.467/2013 and 711.592/2014). All participants signed a statement of informed consent prior to the onset of the data collection process, and the care recipients that were completely dependent (13.5%) were consented by the legal guardian, who was predominantly represented by the caregiver.

The data were entered in a double-blinded dataset in MS Excel 2010. The SPSS program version 21.0 (IBM, Chicago, Illinois, USA) was used for the data analysis. The data presented adherence to normality, as verified by the Kolmogorov-Smirnov test, and therefore parametric statistical tests were run. Descriptive statistics were performed to describe the sample (Table 1). Internal consistency of the ZBI-12 was measured using Cronbach’s alpha, considering α > 0.8 to be indicative of very good to excellent internal consistency. Correlation coefficients were calculated for the item-item and item-score evaluations to determine the strength of the internal correlation of each item. Pearson’s correlation coefficients were calculated to analyze the strength of correlations between the ZBI-12 items and total GDS, PSS and L&B scale scores for the determination of the standard reference (Table 2). To suggest a cut-off point for the ZBI-12, the PSS scores were divided into quartiles and four groups created. For each group, the mean and standard-deviation of ZBI-12 were reported. The mean value on the ZBI-12 for the highest group was the suggested cut-off point (Table 3). One-way ANOVA with Tukey’s post hoc test was used for the comparison of mean ZBI-12 scores according to the PSS quartiles. A p-value ≤ 0.05 was considered indicative of statistical significance.

**Table 1:** Characteristics of 341 older caregivers in São Carlos, SP, Brazil, 2014.

Variable	% or mean ± standard deviation
**Age (years)**	69.6±7.1
60-69	57.8
70-79	30.8
≥80	11.4
**Sex**	
Male	23.2
Female	76.8
**Education (years)**	3.8±3.5
<1	17.6
1-4	62.8
5-8	9.7
≥9	9.7
**Relationship to care recipient**	
Spouse	85.0
Son/daughter	7.3
Son-/daughter-in-law	2.1
Sibling	3.8
Other	1.8
**PSS (points)**	18.5±9.9
**GDS (points)**	3.7±2.7
0-5 (normal)	78.0
6-10 (mild)	18.8
≥ 11 (severe)	3.2
**IADL (care recipient)**	13.7±4.0
Completely dependent	13.5
Partially dependent	86.5

PSS: perceived stress scale; GDS: geriatric depression scale; IADL: instrumental activities of daily living

**Table 2:** Correlation between ZBI items and overall scores on other assessment tools, São Carlos, SP, Brazil, 2014 (n=341).

Items on ZBI-22	Items on ZBI-12	GDS			PSS			Lawton & Brody IADL	
		r	rank		r	rank		r	rank
2.	1.	0.20**	11		0.36**	4		-0.31**	1
3.	2.	0.29**	5		0.39**	1		-0.18**	6
5.	3.	0.30**	4		0.31**	7		-0.04	11
6.	4.	0.23**	9		0.22**	11		-0.06	10
9.	5.	0.31**	3		0.31**	6		0.00	12
10.	6.	0.32**	2		0.38**	2		-0.23**	3
11.	7.	0.24**	8		0.29**	9		-0.11*	8
12.	8.	0.22**	10		0.30**	8		-0.26**	2
17.	9.	0.34**	1		0.36**	3		-0.19**	5
19.	10.	0.26**	7		0.23**	10		-0.11*	7
20.	11.	0.07	12		0.15**	12		-0.09	9
22.	12.	0.27**	6		0.35**	5		-0.20**	4
Total score		0.43**	-		0.53**	-		-0.28**	-

** p<0.01;

* p<0.05; ZBI-12: Zarit Burden Interview - 12-item version; PSS: perceived stress scale; GDS: geriatric depression scale; IADL: instrumental activities of daily living

**Table 3:** Mean±standard deviation of overall 12-Item Zarit Burden Interview score and analysis of variance for Perceived Stress Scale, São Carlos, SP, Brazil, 2014 (n=341).

	1^st^ quartile	2^nd^ quartile	3^rd^ quartile	4^th^ quartile	F statistic (p-value)
PSS*	2.9±3.4 (n=84)	6.0±6.2 (n=89)	7.0±7.3 (n=83)	13.4±10.6 (n=85)	30.55 (< 0.001)

* one-way ANOVA with Tukey's post hoc test: 1^st^ quartile < 2^nd^ quartile < 3^rd^ quartile < 4^th^ quartile; PSS: perceived stress scale.

## RESULTS

Of the 341 caregivers, 57.8% were between 60 and 69 years of age, 76.8% were women and the majority had a low level of education (Table 1). The caregivers provided care for an average of 6.1±4.8 hours per day and did not receive any financial/material support for the costs related to care (83.9%) or any emotional/affective (53.9%) support from other family members or friends for care. Most caregivers had provided care for less than five years (75.2%).

The profile of the care recipient was typically male (70.4%) and aged 73.6±8.5 years (38.7% between 60 and 69 and 39.0% between 70 and 79 years). Mean education was 3.4±3.6 years (56.2% had one to four years of study and 25% never went to school), which is similar to that of the caregivers.

Table 2 shows the correlations between the ZBI-12 items and the other assessment tools administered. The strongest correlations were with the PSS, with all ZBI-12 items correlated at the 99% significance level. Therefore, the PSS was considered the standard reference. Based on the mean position in the ranking, Items 2 (“Do you feel stressed between caring for [care recipient] and trying to meet other responsibilities for your family or work?”), 6 (“Do you feel your health has suffered because of your involvement with [care recipient]?”) and 9 (“Do you feel you have lost control of your life since [care recipient]’s illness?”) were the most strongly correlated with the other assessment tools, whereas Items 4 (“Do you feel that [care recipient] currently affects your relationships with other family members or friends in a negative way?”) and 11 (“Do you feel you should be doing more for [care recipient]?”) had the weakest correlations, which is in agreement with the validation study of the original short version of the ZBI.

The mean of the internal correlations among the items was 0.28 (range 0.03 to 0.67). The item-total score correlation ranged from 0.40 to 0.67. Item 11 had the weakest correlation and Items 2 and 6 had the strongest correlations. Cronbach’s alpha demonstrated very good internal consistency (α=0.81).

Table 3 displays the mean values on the scale for each group stratified by quartiles according to the PSS. The mean scores among the groups differed significantly (ANOVA), with higher ZBI-12 scores found among individuals subdivided in quartiles of PSS. Based on the highest quartile of PSS, a cut-off of 13 points on the ZBI-12 can be suggested for use when screening burden in community-dwelling older caregivers in Brazil.

## DISCUSSION

In the present study, an analysis was performed of the internal consistency and validity of the ZBI-12 administered to older caregivers of community-dwelling older adults in a medium-sized city in Brazil. Cronbach’s alpha (α=0.81) was close to that found by Scazufca^21^ for the original scale (α=0.87) and similar to that of the 12-item scale proposed by Bedard et al.^11^ in one of the follow-up evaluations (α=0.83), as well as the validation study for the short version conducted with 241 caregivers of individuals with dementia at 20 research centers in Spain and Portugal (α=0.89).^26^

Items 11 and 4 had weak correlations with the standard reference assessment tool. A study conducted in a Chinese population of 500 caregivers of individuals with dementia in Hong Kong found the short scale easy to administer, concise, reliable and valid (α=0.81), and also found that Item 4 had a weak correlation to standard reference assessment tools.^27^ Items 6 and 2 had the strongest correlation with the standard reference, which is similar to findings described in a study involving 270 caregivers of individuals with dementia in Thailand, in which Item 2 had a very strong correlation and Item 6 had a moderate correlation and high Cronbach’s alpha.^28^

The cut-off point suggested in the present study for the ZBI-12 for community-dwelling older caregivers was 13. The authors who developed the short version indicated a cut-off point of 17 based on the quartile with the highest scores in the sample, but point out that the data should not be assumed to be normative for the caregiver population.^11^ Moreover, despite being drawn from the community, the sample in the study cited was younger (mean age: 61.01 years) and provided care for individuals with Alzheimer’s disease (72%) and other forms of cognitive deficit (28%), which may explain the higher cut-off point for burden found by the authors.^11^

An investigation that evaluated different short versions of the ZBI in caregivers of patients with cancer, dementia and brain injuries found that the 12-item version had the greatest validity and consistency in the three samples and that the optimum combination of sensitivity and sensitivity on the ROC curve was 92 and 94%, respectively, suggesting a cut-off of 12 points.^29^ Another study in the literature reports that the short version of the ZBI can be administered to caregivers of patients at a specialized clinic for cognitive disorders and that a cut-off point of 17 is coherent for the definition of a high degree of burden; however, mean scores may vary depending on the degree of cognitive decline in the care recipient.^16^

The different cut-off points underscore the need for further studies for the identification of normative data that can be used to define high and low degrees of burden among caregivers in different age groups and who provide care for individuals with different degrees of dependence.

The literature reports factors associated with caregiver burden determined using the ZBI-12. In a study conducted in London with caregivers of patients with lung cancer and heart failure, burden was associated with depression, anxiety, quality of life, caregiver reports of the quality of care and patient wellbeing, caregiver’s sleep quality, whether the caregiver received help from friends and relatives, caregiver’s age, positive care experiences and dysfunctional coping strategies in the bivariate analysis. After the regression analysis, burden was associated with low quality patient care and worse psychological health of the caregiver.^30^ In a study involving informal caregivers of older adults with different degrees of cognitive impairment, burden was associated with depressive symptoms, behavioral problems and impaired activities.^32^

Studies that evaluate predictors of burden in caregivers of older adults with dementia reveal that behavioral symptoms of the care recipient are among the factors that increase the level of burden in the caregiver.^33^ Brazilian researchers used the ZBI to compare the degree of burden in 20 caregivers of older adults divided into two groups of ten. In both groups, the care recipients were bedridden and highly dependent, but only one group of patients had dementia. The mean score for the caregivers of older adults without dementia was 30.4 and the score for caregivers of older adults with dementia was, on average, 18.8 points higher, a statistically significant difference (p=0.006).^34^ A higher degree of burden in caregivers of older adults with dementia has also been identified in international studies that used the ZBI-12. In a study conducted with 194 caregivers of individuals with dementia in different age groups that resided in rural communities in Canada, the researchers found a cut-off point of 14.19 for the evaluation of burden.^15^ The study in which the ZBI-12 originated involved a sample of caregivers of older adults, the majority of which had dementia, and found a cutoff point of 17.^11^ Differences in the care context and populations may contribute to different degrees of caregiver burden, which underscores the need for studies that establish a cut-off point for the evaluation of burden using the ZBI-12, specifically for caregivers of older adults with dementia.

Despite having different definitions, with burden related specifically to providing care^9^^,^ 21 and stress related to general life situations, the two concepts are strongly correlated. This strong correlation has been described in previous studies that employed the same assessment tools (PSS and ZBI) for caregivers of community-dwelling individuals with dementia,^35^^,^ 36 as well as caregivers of community-dwelling older adults.^37^ This correlation has also been described when using other assessment tools for caregivers treated in primary care.^38^ Moreover, a meta-analysis that identified determinant models of caregiver burden found that the mental and physical health of the caregiver were associated with burden and that depression and other mental health-related outcomes in the caregiver can be consequences of burden.^39^

The degree of burden related to the demands of daily care provided to a dependent older adult is important to evaluate due to the negative influence exerted on physical, psychological and cognitive health and has driven the development of care models directed at caregivers themselves.^7^^,^ 40 ^-^ 42 In Brazil, the majority of studies that evaluate burden in family caregivers of community-dwelling older adults use the ZBI.

A meta-analysis, using a sample of dementia caregivers, compared the diagnostic utility of the various short versions of the Zarit Burden Interview (ZBI) with the original scale and identified that the 6-item, 7- item and both 12-item versions of the ZBI are equivalent to the original 22-item version in terms of diagnostic utility. These shorter versions can be utilized to assess caregiver burden with greater convenience and reliability.^17^

Studies evaluating caregiver burden using the ZBI-12 have been conducted with different age groups. In a study conducted in Australia to evaluate the impact of the behavior of patients with amyotrophic lateral sclerosis and motor disability on caregiver burden, most caregivers were women (69.3%) and mean age was 60.8 ± 12 years.^31^ In a Belgian study exploring aspects associated with burden in informal caregivers of older adults with cognitive impairment, mean age was 60.9 ±13.3 years.^32^ In the present study, however, the sample was composed exclusively of older caregivers.

This study has particular importance for the identification of burden in community-dwelling older caregivers. A fast, effective evaluation of the perceived burden of caregivers especially in the primary care context in Brazil, can lead to the identification of the older caregiver population at risk of complications and adverse outcomes.^41^ ^-43^ However, this study is not without limitations, the sample was composed only of older caregivers with specific characteristics, which limits the generalization of the findings and the ZBI-12 cutoff. This sample comprised older female and male caregivers in an inner city community of Brazil. They were supporting for their loved ones in at least on dependence of activity of daily living and the results should be interpreted considering this profile. Nonetheless, the results can serve as a starting point for reflections and further studies involving the ZBI-12.

The present study analyzed the internal consistency and validity of the ZBI-12 administered to older caregivers of dependent older adults living in the community. The results show that the measure has good internal consistency and can be considered valid for this population. Moreover, a cut-off point of 13 is recommended for the determination of a high degree of burden, but should not be assumed as normative data. These findings can serve as the basis for subsequent studies that seek to suggest data for caregivers in different age groups that provide care to individuals with different degrees of dependence.
